# Liver Abscess Secondary to Crohn’s Disease: A Case Report

**DOI:** 10.7759/cureus.23157

**Published:** 2022-03-14

**Authors:** Ariana R Tagliaferri, Heemani Ruparel, Gabriel Melki, Walid Baddoura

**Affiliations:** 1 Internal Medicine, St. Joseph's Regional Medical Center, Paterson, USA; 2 Medicine, St. Joseph's Regional Medical Center, Paterson, USA; 3 Gastroenterology, St. Joseph's Regional Medical Center, Paterson, USA

**Keywords:** gastroenterology, crohn’s disease, inflammatory bowel disease, autoimmune disease, liver abscess

## Abstract

Crohn’s disease (CD) is a type of inflammatory bowel disease (IBD) and extra-intestinal manifestations are common. Although common features of CD include fistulation and abscess formation, they typically manifest exclusively in the lower gastrointestinal (GI) tract and in patients who do not have adequate control over their disease. Pyogenic liver abscess is rare in the general population and is an unusual and extra-intestinal manifestation of CD. Herein, we present a patient with Crohn’s ileo-colitis who presented with generalized abdominal pain and fevers and was found to have multiple pyogenic liver abscesses biopsy-proven to be secondary to CD. The patient’s liver abscesses were refractory to repeated CT-guided drainage and antibiotic therapy. This paper illustrates a rare condition in the general population and those with CD. We intend to discuss the differences of pyogenic liver abscesses in CD compared to the general population, the rarity of this presentation and propose a unique mechanism by which the patient may have developed this liver abscess. It is common for clinicians to mistake the diagnosis of febrile illness with or without abdominal pain as a simple reactivation of CD, and thus it is important to keep pyogenic liver abscess on the differential even if their disease state is otherwise well controlled.

## Introduction

This article was previously presented as a poster at the American College of Gastroenterology Annual Scientific Meeting in Las Vegas in 2021.

Crohn’s disease (CD) is characterized by discontinuous lesions along the gastrointestinal (GI) tract consisting of transmural inflammation with lymphocytic infiltration [1]. The etiology of CD is still poorly understood, but genetic and environmental factors are well known in its pathogenesis. CD is the result of an inappropriate inflammatory response that results in chronic intestinal damage, as well as serious life-threatening complications [1,2]. The prevalence of CD in the North American and European population is estimated to be about 250-300 per 100,000 people [1]. Commonly associated risk factors include age, family history in a first-degree relative, history of smoking, and living in urban areas [2]. Features of the disease include fistulas, abscesses, perianal disease and GI involvement in other sites of the GI tract [3]. CD is known to have extra-intestinal manifestations including arthropathy, uveitis, skin disorders, primary sclerosing cholangitis, pulmonary involvement, renal stones, and secondary amyloidosis [3]. Specifically, a pyogenic liver abscess is an uncommon extra-intestinal complication accounting for about 3-5 per 100,000 hospital admissions, but it is an important manifestation to consider due to the associated high mortality [4]. Pyogenic liver abscesses are highly under-recognized [4].

Liver abscesses can develop from fistulas between loops of the intestine or from an underlying intraabdominal infection [5]. Most cases of liver abscesses are seen in young patients with long-standing and uncontrolled CD. Persistent transmural inflammation can further facilitate the invasion of microorganisms to the liver [5]. These factors in conjunction with other factors such as fistula formation, long-term steroid use, as well as chronic malnutrition increase the risk of developing liver abscesses [5]. It is important for clinicians to consider liver abscess as a differential in a CD patient presenting with symptoms of fever and right upper quadrant, despite having adequate control over their disease [6].

## Case presentation

A 45-year-old Hispanic male with a past medical history of Crohn's ileo-colitis with hemicolectomy, complicated by anal fistulas, presented with abdominal pain in the right upper quadrant, subjective fever of three weeks duration and a visible new peri-rectal fistula upon self-examination [6]. Review of systems was also significant for unintentional 10-pound weight loss over the course of five weeks and increased stool output from his ileostomy lasting a few weeks prior to admission [6]. The patient was compliant with infliximab and 6-mercaptopurine for CD and otherwise had good control over his inflammatory bowel disease (IBD). On admission the patient was febrile (38.4°C), hypotensive (106/72 mmHg), with a heart rate of 86 beats per minute, respiratory rate of 18 and saturation of 100% on room air [6]. He was well appearing, in no acute distress with an abdominal exam notable for moderate tenderness in the right upper quadrant upon superficial and deep palpation. He was noted to have an ileostomy bag filled with normal brown stool and an external anorectal exam showed multiple fistulas draining sero-fibrinous fluid without odor [6]. The remainder of the exam was unremarkable. Labs were significant for leukocytosis with left shift, normocytic anemia, thrombocytosis, mild hyponatremia, mild hyperkalemia, elevated alkaline phosphatase, hypoalbuminemia, hypocalcemia, and acute kidney injury (baseline creatinine 1.3 mg/dL) (Table 1).

**Table 1:** Complete admission labs of the comprehensive metabolic panel (left columns) and complete blood count (right columns).

Comprehensive Metabolic Panel		Complete Blood Count	
Sodium	127 mEq/L	White blood cells	17.6 x10^3/mm3
Potassium	5.3 mEq/L	Hemoglobin	8.8 g/dL
Chloride	101 mEq/L	Red blood cells	3.15 x10^6/mm3
Bicarbonate	14 mEq/L	Hematocrit	29.2 %
Glucose	113 mg/dL	Mean Corpuscular Hemoglobin	27.9 pg
Calcium	8.4 mg/dL	Mean Corpuscular Volume	92.7 fL
Blood urea nitrogen	43 mg/dL	Mean Corpuscular Hemoglobin Concentration	30.1 g/dL
Creatinine	2.97 mg/dL	Red Cell Distribution Width	15.3 %
Total Bilirubin	0.3 mg/dL	Platelet Volume	9.4 fL
Total Protein	8.4 g/dL	Platelets	491 K/mm3
Albumin	3.3 g/dL	Neutrophil Absolute	15.03 x10^3/mm3
Alkaline Phosphatase	118 unit/L	Monocyte Absolute	1.21 x10^3/mm3
Aspartate transaminase	13 unit/L	Basophil Absolute	0.05 x10^3/mm3
Alanine transaminase	13 unit/L	Lymphocyte Absolute	0.65 x10^3/mm3
Lipase	82 unit/L	Eosinophil Absolute	0.01 x10^3/mm3
C-Reactive Protein	181.7 mg/L	Erythrocyte Sedimentation Rate	>140 mm/hr

**Figure 1:**
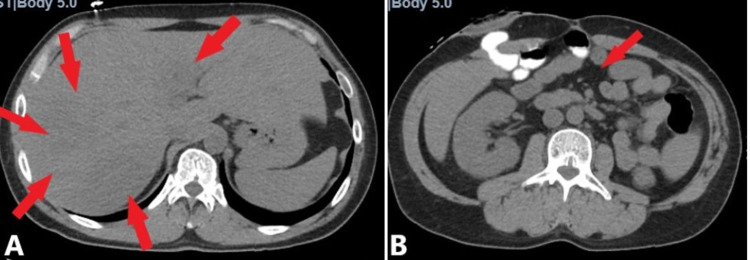
Computerized tomography of the abdomen and pelvis without intravenous contrast. At least five new low-density lesions were identified. (A) Left lobe lesion measuring approximately 1.2 cm (red arrow). Lateral dome right lobe lesion measuring approximately 1.4 cm (red arrow). Posteromedial dome of right lobe lesion measuring approximately 1.4 cm (red arrow). Lateral right lobe lesion measuring approximately 1.4 cm (red arrow). Inferior right lobe lesion measuring approximately 1.0 cm (red arrow). Lesions concerning metastases versus multifocal hepatic abscess. (B) Right upper quadrant ostomy present, with bowel anastomosis in the right mid-abdomen. There is mild mesenteric congestion (red arrow).  Normal size mesenteric lymph nodes are evident.

The urinalysis was positive for small leukocyte esterase, trace bacteria and white blood cells (5-10/HPF). A Computerized Tomography (CT) scan of the abdomen and pelvis without contrast was performed, demonstrating multiple liver lesions highly suspicious of liver abscess with the differential diagnosis including metastasis (Figures 1A, 1B) [6].

**Figure 2:**
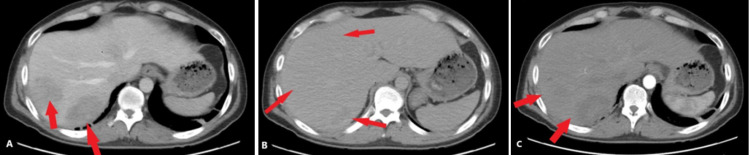
Computerized tomography of the abdomen and pelvis with intravenous contrast. There are two ill-defined hypodense lesions in the right lobe of the liver measuring 6.3 x 4.4 and 5.2 by 4.7 cm and the left lobe 5 x 3 cm (red arrows). The lesions are predominantly hypodense compared to the liver parenchyma on venous phase (A) and arterial phase (C) with non-enhancing central hypodensities. This may represent scar or necrosis. The lesions are predominantly isodense on delayed phase imaging (B). There are few non-enhancing sub-centimeter hypodense lesions in the liver that are too small to characterize and may represent cysts.

The patient was admitted to the hospital for sepsis and was started on intravenous piperacillin-tazobactam 3.375 milligrams every eight hours and intravenous fluids. As there was a concern for infection, the patient’s home medications were stopped. Further workup revealed negative blood and stool cultures; however, the urine culture was positive for *Escherichia coli*. During hospitalization, the fever and pain persisted despite antibiotics and the patient underwent magnetic-resonance cholangiopancreatography (MRCP), which revealed a normal common bile duct without stone or dilatation and liver lesions compatible with abscess formation [6]. The patient underwent a CT-guided aspiration, draining 10 cc of bloody-pus from the posterior right lobe, but fluid cultures came back negative [6]. As the patient clinically improved, he was medically optimized and discharged; however, he returned to the ED one week later with recurrent fevers and was re-admitted for possible necrotic malignancy [6]. A repeat MRCP demonstrated the decreased size of all liver abscesses, consistent with repeat CT imaging with intravenous contrast (Figures 2A-2C) [6].

The patient underwent a repeat CT-guided liver biopsy which showed multiple well-formed noncaseating granulomas in the hepatic lobules, and to a lesser extent within the portal tracts. Both acute and chronic inflammation were noted with multiple foci of microabscesses and granulation tissue [6]. These pathological and histological findings were consistent with CD. The patient continued to improve and was re-started on his home medications prior to discharge and antibiotic therapy was discontinued [6].

## Discussion

Extra-intestinal manifestations are seen in 25%-40% of IBD patients [6,7]. These can occur in any organ system of the body; most commonly ocular, hepato-pancreato-biliary, musculoskeletal and dermatologic systems [7]. Various hepatobiliary diseases are seen in IBD, with primary sclerosing cholangitis being more common in ulcerative colitis (UC), and cholelithiasis and granulomatous hepatitis being more common in CD [6,8]. Specifically, there is a higher incidence of generalized infections, fistulation and abscess formation in CD [8,9]. Although compared to the general population, CD patients have a higher risk of developing liver abscesses, the overall incidence even in this population is rare [8,9]. The first description of a liver abscess in the setting of CD was in 1946 and since then approximately only 18 other cases have been reported [6,8,9].

Liver abscesses are caused by enteric bacteria, such as *Streptococci *or *E. coli *[6,8]. Many patients in the general population respond to antibiotic therapy [8]. Liver abscesses present and manifest differently in those with CD compared to the general population [6,8]. A retrospective review assessed 18 cases of patients with pyogenic abscesses in the setting of IBD [6,8]. Fever was the most common presenting symptom, complicating the differential and delaying diagnosis [8]. Only 50% of the patients presented with abdominal pain [6,8]. Compared to the general population, CD patients with pyogenic abscesses present at a younger age and are more likely to have multiple abscesses rather than a solitary lesion [6,8]. These patterns are consistent with our patient’s presentation, as he was 45 years old and had multiple lesions on initial imaging [6]. Other studies have demonstrated that CD patients do not respond to medical management alone, warranting surgical/percutaneous intervention [6,9]. Our patient required multiple drainages and was refractory to both medical and surgical management, prior to clinical improvement [6]. It is likely that given the recurrent fevers, worsening diarrhea and mild bowel edema noted on imaging, he may have had an underlying intraabdominal infection predisposing him to abscess formations in the liver [6]. It is also important to note that our patient also did not have a history of hepatobiliary disease.

## Conclusions

It is common for clinicians to mistake the diagnosis of febrile illness with or without abdominal pain as a simple reactivation of CD. It is possible that liver abscesses may be underdiagnosed. This case is now one of 19 known cases of pyogenic liver abscesses in the setting of CD, and his case is particularly unique because the patient had no prior history of extra-intestinal manifestations apart from previous perianal disease. Additionally, his IBD was well controlled under his current medication regimen. Thus, it is imperative for clinicians to consider hepatic pyogenic abscess on the differential when CD patients present with non-specific complaints.
